# Mechanistic Insights into the Cardioprotective Effects of Mesenchymal Stem Cell-Derived Exosomes in Myocardial Ischemic Injury: A Systematic Review

**DOI:** 10.3390/pharmaceutics18030346

**Published:** 2026-03-11

**Authors:** Nur Athirah Othman Basri, Nur Aishah Che Roos, Amilia Aminuddin, Adila A. Hamid, Chua Kien Hui, Mohd Kaisan Mahadi, Jaya Kumar, Azizah Ugusman

**Affiliations:** 1 Department of Physiology, Faculty of Medicine, Universiti Kebangsaan Malaysia, Kuala Lumpur 56000, Malaysia; p131665@siswa.ukm.edu.my (N.A.O.B.); amilia@hctm.ukm.edu.my (A.A.); adilahamid@hctm.ukm.edu.my (A.A.H.); jayakumar@ukm.edu.my (J.K.); 2 Faculty of Health Sciences, MAIWP International University, Kuala Lumpur 68100, Malaysia; 3 Faculty of Medicine and Defence Health, National Defence University of Malaysia, Kuala Lumpur 57000, Malaysia; nuraishah@upnm.edu.my; 4 Cardiovascular and Pulmonary (CardioResp) Research Group, Universiti Kebangsaan Malaysia, Bangi 43600, Malaysia; 5 Supergenics Berhad, Subang Jaya 47650, Malaysia; ckienhui@gmail.com; 6 Centre for Drug and Herbal Development, Faculty of Pharmacy, Universiti Kebangsaan Malaysia, Jalan Raja Muda Abdul Aziz, Kuala Lumpur 50300, Malaysia; kaisanmahadi@ukm.edu.my

**Keywords:** extracellular vesicles, exosomes, hypoxia/reoxygenation, ischemic heart disease, mesenchymal stem cells, myocardial ischemia/reperfusion injury

## Abstract

**Background:** Myocardial ischemic injury, encompassing acute myocardial infarction (MI) and ischemia/reperfusion (I/R) injury, remains a major cause of cardiac morbidity and mortality worldwide, and is driven by interconnected molecular and cellular processes, including cardiomyocyte apoptosis, inflammatory activation, mitochondrial dysfunction, oxidative stress, and impaired angiogenesis. Mesenchymal stem cell (MSC)-derived exosomes have emerged as a promising cell-free nanotherapeutic strategy for cardiac repair due to their ability to transfer bioactive molecules that modulate multiple signaling networks involved in myocardial survival and regeneration. This systematic review aimed to synthesize evidence on the mechanistic basis of MSC-derived exosome mediated cardioprotection in myocardial ischemic injury. **Methods:** A systematic search of Ovid MEDLINE, Scopus, and Web of Science was conducted to identify studies investigating the effects of MSC-derived exosomes on myocardial ischemic injury. Eligible studies included clinical and preclinical models of MI or I/R injury assessing functional, biochemical, and molecular outcomes. **Results**: Seven preclinical studies published between 2015 and 2025 met the inclusion criteria. Exosome administration consistently improved cardiac function, reduced infarct size, and preserved myocardial architecture. Biochemical analyses revealed decreased cardiac injury markers, alongside suppressed apoptosis, inflammation, and oxidative stress. Mechanistically, MSC-derived exosomes delivered regulatory miRNAs (e.g., miR-19a, miR-125b, miR-205, miR-294) and lncRNAs (HAND2-AS1) that modulated key signaling pathways including PI3K/Akt, JAK2/STAT3, HAND2-AS1/miR-17-5p/Mfn2, and HIF-1α/VEGF. These molecular effects collectively inhibited apoptotic and inflammatory responses, enhanced mitochondrial integrity, and promoted angiogenesis and myocardial repair. **Conclusions:** MSC-derived exosomes confer robust cardioprotection against myocardial ischemic injury through integrated anti-apoptotic, anti-inflammatory, antioxidant, and pro-angiogenic mechanisms. Their multifaceted bioactivity, low immunogenicity, and potential for targeted delivery highlight their potential as a next-generation nanomedicine for ischemic heart disease. Future studies should emphasize standardized exosome production, mechanistic profiling, and translational validation in large-animal and clinical models.

## 1. Introduction

Myocardial ischemic injury, encompassing both acute myocardial infarction (MI) and ischemia/reperfusion (I/R) injury, remains a major cause of morbidity and mortality worldwide. While timely reperfusion after infarction salvages jeopardized myocardium, the abrupt restoration of blood flow can paradoxically precipitate further damage, a phenomenon known as I/R injury [1,2]. Reperfusion initiates a cascade of pathological events, including oxidative burst, calcium overload, mitochondrial dysfunction, endothelial activation, and sterile inflammation, thereby amplifying myocyte death and predisposing to adverse remodeling, heart failure, and arrhythmias [3,4]. Increasing evidence identifies mitochondrial permeability transition pore (mPTP) opening, excessive reactive oxygen species (ROS) production, and dysregulated immune activation as central and interdependent drivers of reperfusion-associated myocardial injury, underscoring the need for multi-target therapeutic strategies rather than single-pathway interventions [5,6].

Despite advances in revascularization, pharmacotherapy, and mechanical support, no clinically validated therapy consistently mitigates ischemia-induced myocardial injury [4]. Although antioxidant and anti-inflammatory approaches have shown promising outcomes in experimental models, their failure to translate into consistent clinical benefits highlights a persistent translational gap in cardioprotection research [7].

Against this backdrop, mesenchymal stem cells (MSCs) have emerged as a promising regenerative strategy. However, accumulating evidence indicates that their therapeutic effects are predominantly paracrine, mediated through the secretion of extracellular vesicles (EVs), commonly referred to as exosomes [8,9]. Limited MSC retention and engraftment within injured myocardium further support the concept that reparative effects arise primarily from secreted bioactive factors rather than direct cellular replacement [10,11]. These nanosized vesicles (approximately 30–150 nm) transport bioactive cargo of proteins, lipids, and RNAs, including microRNAs, which modulate recipient cell survival, immune responses, and tissue repair [12].

Exosomes originate from the endosomal pathway via inward budding of multivesicular bodies and exhibit intrinsic stability, low immunogenicity, and the ability to traverse biological barriers, rendering them attractive candidates for cell-free therapeutic applications [13,14]. In preclinical models of MI and I/R injury, MSC-derived exosomes have consistently reduced infarct size, preserved left ventricular function, and attenuated apoptosis and inflammation [15,16,17,18]. More recently, large-animal and translational studies have demonstrated improvements in ventricular remodeling and microvascular density, extending their therapeutic relevance beyond small rodent models [19,20].

Mechanistically, MSC exosomes deliver miRNA and protein cargo that converge on canonical pro-survival and reparative signaling pathways. Reported mechanisms include activation of phosphatidylinositol 3-kinase (PI3K)–protein kinase B (Akt)/extracellular signal-regulated kinase (ERK) signaling, components of the reperfusion injury salvage kinase (RISK) pathway, mitochondrial protection, inhibition of caspase-mediated apoptosis, attenuation of nuclear factor kappa-B (NF-κB)-driven inflammation, and promotion of angiogenesis [15]. Emerging evidence further highlights extensive crosstalk between PI3K/Akt signaling, Nrf2-mediated antioxidant pathways, and mitochondrial dynamics, suggesting coordinated network regulation rather than isolated molecular events [21].

Representative miRNAs implicated in cardioprotection include miR-21, miR-182, miR-125b, miR-19a, and miR-25-3p, which target signaling axes such as phosphatase and tensin homolog (PTEN)/Akt, c-Jun N-terminal kinase (JNK)/caspase-3, janus kinase 2 (JAK2)/signal transducer and activator of transcription 3 (STAT3), and hypoxia-inducible factor 1-alpha (HIF-1α)/vascular endothelial growth factor (VEGF) to improve cardiomyocyte survival and microvascular regeneration [15,22]. Beyond individual miRNAs, recent studies emphasize the synergistic action of multiple exosomal cargos, including long non-coding RNAs and circular RNAs, which further fine-tune inflammatory and apoptotic signaling in ischemic myocardium [23]

Consistent with these molecular effects, preclinical studies report improvements in oxidative stress markers such as malondialdehyde (MDA), superoxide dismutase (SOD), and reduced glutathione (GSH), alongside reductions in cardiac injury biomarkers including creatine kinase–myocardial band (CK-MB) and troponin, accompanied by functional recovery [24]. Additionally, modulation of macrophage polarization from a pro-inflammatory M1 phenotype toward a reparative M2 phenotype has emerged as a key immunomodulatory mechanism underlying MSC-exosome-mediated cardioprotection [25].

Despite accumulating evidence, findings remain fragmented due to heterogeneity in experimental models, exosome sources, isolation techniques, dosing regimens, timing of administration, and outcome measures [26,27]. This variability limits direct cross-study comparability and obscures consistent mechanistic patterns. Although optimization of dosing strategies and timing represents an important translational objective, the primary aim of the present review is to systematically synthesize and integrate molecular signaling pathways underlying MSC-derived exosome-mediated cardioprotection.

To date, mechanistic insights, particularly regarding how exosomes coordinate anti-apoptotic, anti-inflammatory, antioxidant, and angiogenic pathways, have largely been presented in descriptive or narrative formats [28,29]. A Preferred Reporting Items for Systematic Reviews and Meta-Analyses (PRISMA)-guided synthesis specifically focused on mechanistic signaling cascades in myocardial ischemic injury remains limited. Accordingly, a systematic integration of these pathways is warranted to clarify recurring mechanistic hubs and regulatory networks that consistently mediate MSC-derived exosome-mediated myocardial repair.

This systematic review therefore synthesizes and critically appraises available preclinical and clinical evidence on the role of MSC-derived exosomes in myocardial ischemic injury, including both MI and I/R models. In addition to summarizing therapeutic outcomes, the review examines convergent signaling pathways, particularly miRNA-mediated PI3K/Akt [30] and related networks across studies to highlight recurring molecular mechanisms with potential translational relevance. Importantly, the primary focus is on molecular and cellular mechanisms rather than on optimization of dosing regimens, delivery routes, or clinical implementation strategies. Collectively, this review provides an integrated mechanistic framework to inform hypothesis-driven translational development of cell-free therapies for myocardial ischemic injury.

## 2. Methods

This study was conducted in accordance with the PRISMA 2020 guidelines (Supplementary Materials), ensuring a systematic and transparent process for literature searching, data extraction, and evidence synthesis [31]. The protocol was registered at the International Platform of Registered Systematic Review and Meta-analysis Protocols (INPLASY2024110023) [32].

### 2.1. Search Strategy

A comprehensive search was conducted in Ovid MEDLINE, Scopus, and Web of Science using a strategy that combines terms related to exosomes, MSC, and myocardial ischemic injury. The provisional string was (exosome) AND (mesenchymal stem cells OR mesenchymal stromal cells) AND (myocardial ischemia OR myocardial infarction OR cardiac infarction OR myocardial ischemia reperfusion* OR ischemia reperfusion OR ischemic heart disease OR hypoxia reoxygenation OR myocardium OR cardiomyocyte OR hypoxia OR reperfusion OR reoxygenation). Titles and abstracts were screened based on the PICOS framework relevant to MSC-derived exosomes and myocardial ischemic or I/R injury. No geographical or publication date limits were applied, and only English-language publications were included. The reference lists of included studies were also hand-searched to identify additional records [32]. All databases were searched from inception until 26 July 2025.

### 2.2. Inclusion and Exclusion Criteria

Eligibility was prespecified using the PICOS framework to identify studies evaluating the independent therapeutic effects of unmodified MSC-derived exosomes in MI or I/R injury, which share overlapping pathophysiological mechanisms of myocardial ischemic injury. Eligible populations included preclinical in vivo MI or I/R models and in vitro hypoxia/reoxygenation (H/R) cardiomyocyte models. Clinical studies were eligible if they investigated acute MI or myocardial I/R injury. Studies were excluded if they were non-original publications (reviews, editorials, or conference abstracts), involved chronic or stable ischemic heart disease (IHD) without an acute ischemic component, non-ischemic heart failure models, or chronic post-MI remodeling without an acute insult. Studies focusing on non-cardiac organs were also excluded [32].

The intervention of interest was administration of unmodified MSC-derived exosomes via any route. All exosome sources, isolation methods, doses, administration routes, and treatment durations were eligible. However, studies using modified exosomes (e.g., drug-loaded or material-conjugated) or combining pharmacological treatments with exosome administration were excluded to ensure evaluation of the independent effects of MSC-derived exosomes. Comparators included no treatment, placebo, standard therapy, or conventional reperfusion care.

Primary outcomes included infarct size, while secondary outcomes comprised cardiac function indices (e.g., ejection fraction, fractional shortening), cardiomyocyte viability, and cardiac injury biomarkers (e.g., lactate dehydrogenase (LDH), CK-MB). These criteria were applied strictly during full-text screening to ensure inclusion of studies specifically investigating the independent mechanistic effects of MSC-derived exosomes in acute myocardial ischemic or I/R models.

### 2.3. Study Selection

Following de-duplication, two reviewers (N.A.O.B. and A.U.) independently screened the titles and abstracts for relevance to MSC-derived exosomes and myocardial ischemia or I/R injury. Potentially eligible records were then assessed in full text against the predefined inclusion and exclusion criteria. Any disagreements were resolved through discussion, and a third reviewer (A.A.H.) was consulted to reach consensus when necessary. The reference lists of the included articles were also screened to identify additional eligible records.

### 2.4. Data Extraction

Two reviewers (N.A.O.B. and A.U.) independently extracted data using a predefined, standardized form, with discrepancies resolved through discussion or adjudication by a third reviewer (A.A.H.). Data were compiled in Microsoft Excel and included study characteristics (first author, year, title, country, and design), intervention details (population/model, exosome source, route and timing of administration, treatment duration, and dose), comparators (no treatment, standard therapy, or placebo), and outcomes of interest according to the PICOS framework.

### 2.5. Risk of Bias Assessment

Risk of bias (RoB) assessed independently by two reviewers (N.A.O.B., A.U.), with disagreements resolved by discussion or by a third reviewer (A.A.H.). For animal studies, the SYRCLE RoB tool was used to evaluate selection bias (random sequence generation, baseline characteristics, allocation concealment), detection bias (random housing, blinding, random outcome assessment), attrition bias (incomplete outcome data), reporting bias (selective reporting), and other potential sources of bias [33]. Each domain was rated as having low, unclear, or high risk of bias. The RoB assessment was not used as an eligibility criterion.

## 3. Results

### 3.1. Study Selected

**Figure 1:**
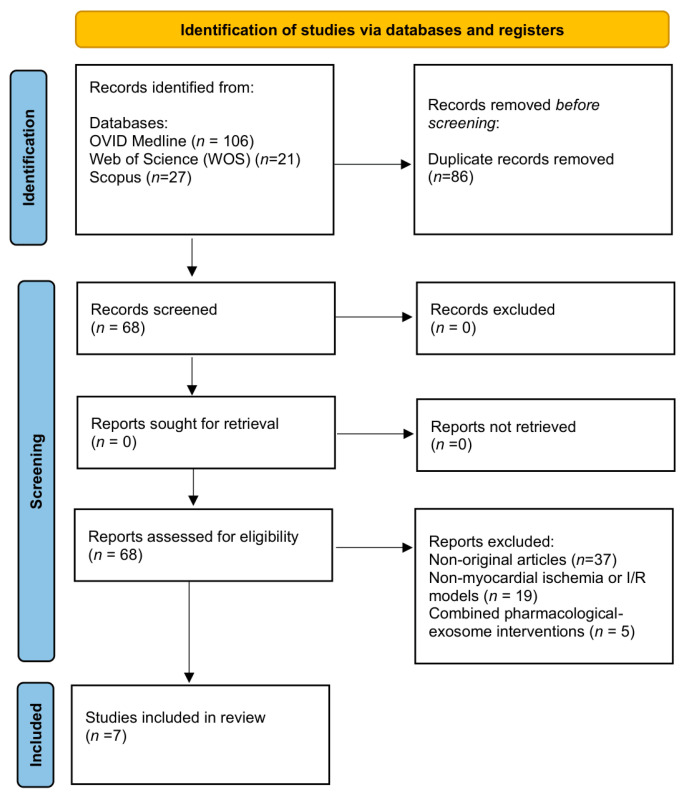
Preferred Reporting Items for Systematic Reviews and Meta-Analyses (PRISMA) 2020 flow diagram for the systematic review. I/R = ischemia/reperfusion.

A total of 154 records were identified across three databases (Ovid MEDLINE, *n* = 106; Scopus, *n* = 27; Web of Science, *n* = 21). After removal of 86 duplicates, 68 records remained for screening. No additional records were identified through citation searching. Of these, 61 studies were excluded for predefined reasons: 37 were non-original publications (reviews, editorials, or conference abstracts), 19 did not investigate myocardial ischemia or I/R injury models, and five involved combined pharmacological–exosome interventions that precluded evaluation of the independent effects of native MSC-derived exosomes. Seven studies [3,4,12,34,35,36,37] met all prespecified inclusion criteria. Eligible studies were original in vivo or in vitro investigations of unmodified MSC-derived exosomes administered in myocardial ischemic injury models, with clearly defined functional and molecular outcomes. These seven studies were included in the final qualitative synthesis. The study selection process is illustrated in Figure 1.

### 3.2. Risk of Bias

**Figure 2:**
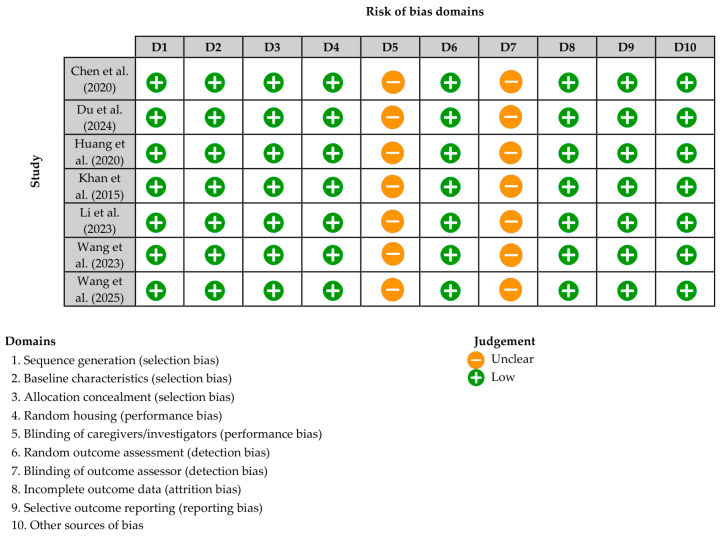
Systematic Review Center for Laboratory Animal Experimentation (SYRCLE) risk of bias summary of the included studies [3,4,12,34,35,36,37].

Overall, most SYRCLE domains (D) were rated as having a low risk of bias across all included studies, including sequence generation (D1), baseline characteristics (D2), allocation concealment (D3), random housing (D4), random outcome assessment (D6), incomplete outcome data (D8), selective reporting (D9), and other sources of bias (D10). However, both blinding domains, namely blinding of caregivers/investigators (D5) and blinding of outcome assessors (D7), were consistently rated as unclear due to insufficient reporting. No domain was judged to be at high risk in any study. While the overall methodological profile suggests acceptable internal validity, the absence of clearly reported blinding procedures may introduce potential performance and detection bias. A detailed summary is presented in Figure 2.

### 3.3. Study Characteristics

Seven preclinical studies published between 2015 and 2025 were included, all evaluating the cardioprotective effects of MSC-derived exosomes in models of myocardial ischemic injury (Table 1). Of these, six studies combined in vivo and in vitro experiments, while one study utilized an in vitro model only [3]. The in vivo studies primarily employed rat or mouse models of acute MI or I/R injury induced by ligation of the left anterior descending (LAD) coronary artery. The in vitro models involved cell-based hypoxia/reoxygenation (H/R) injury assays using H9c2 cardiomyocytes [3,4,12,34,35,36,37], neonatal rat cardiomyocytes (NRCMs) [37], human embryonic stem cell (hESC)-derived cardiomyocytes [35], cardiac progenitor cells (CPCs) [36], and endothelial cells, including human umbilical vein endothelial cells (HUVECs) [35], and human microvascular endothelial cells (HMEC-1) [37]. In one study, lipopolysaccharide (LPS)-stimulated RAW 264.7 macrophages were used to investigate underlying molecular mechanisms [4].

Exosomes were isolated from bone marrow MSCs (BMSCs) [3], adipose-derived MSCs (ADSCs) [37], human umbilical cord MSCs (hucMSCs) [38], and murine embryonic stem cells (mESCs) [36]. In animal models, exosomes were administered intravenously or intramyocardially at doses ranging from 50 µg to 400 µg/g. In in vitro experiments, exosomes were added to culture media at concentrations of 5–100 µg/mL. The timing of exosome administration varied across studies (Table 1). In in vivo models, exosomes were delivered pre-ischemia (2 h before LAD ligation), during sustained ischemia (immediately or 25 min after LAD ligation), or at reperfusion onset. In vitro studies most commonly administered exosomes at the onset of reoxygenation in H/R models, although some employed treatment during hypoxia, co-treatment during oxidative stress, or post-injury ex vivo exposure. This variability in treatment window represents an additional source of heterogeneity across experimental designs.

### 3.4. Effects of MSC-Derived Exosomes on Myocardial Ischemic Injury

Across the included studies, treatment with MSC-derived exosomes produced consistent and significant cardioprotective outcomes in both animal and cell-based models of myocardial ischemic injury. In vivo, exosome-treated animals exhibited improved cardiac performance, evidenced by increased left ventricular ejection fraction (LVEF), systolic pressure (LVSP), fractional shortening (LVFS) and wall motion (LVWM), and decreased left ventricular end-diastolic pressure (LVEDP), end-systolic dimension (LVESD) and end-diastolic dimension (LVEDD) compared with untreated controls [34,35,36,37,39]. Correspondingly, histopathological assessments consistently revealed smaller infarct areas, preserved myocardial fibers, and diminished fibrotic changes and inflammatory cell infiltration in treated hearts [4,34,35,36,37,39].

Biochemical analysis further supported these findings, with exosome-treated animals and cardiomyocytes showing reduced cardiac injury markers, including LDH, CK-MB, and cardiac troponin T (cTnT) [4,34]. In vitro, MSC-derived exosomes enhanced cardiomyocyte viability, proliferation and migration, and reduced apoptotic cells following H/R exposure [12,34,35,37,40]. These biochemical, structural, and functional improvements conferred by MSC-derived exosomes involved multiple mechanisms, including the regulation of apoptosis, inflammation, oxidative stress, angiogenesis and myocardial repair.

### 3.5. Anti-Apoptotic Mechanisms of MSC-Derived Exosomes

Collectively, the evidence indicates that MSC-derived exosomes inhibit cardiomyocyte apoptosis through multiple interrelated molecular pathways. Their protective effects are primarily mediated by the transfer of miRNAs, including miR-19a,20 miR-125b,19 miR-205 [37], and miR-294 [36], as well as long non-coding RNA (lncRNA) such as heart- and neural crest derivative-expressed 2-antisense RNA 1 (HAND2-AS1) [3], which converge on the JAK2/STAT3 and PI3K/Akt signaling cascades to suppress apoptosis. By downregulating pro-apoptotic mediators (Bax, caspase-3, FasL) and upregulating survival-associated proteins (Bcl-2), these exosomes preserve cardiomyocyte viability and maintain myocardial structural integrity following ischemic injury [3,12,34,35,37].

Treatment with BMSC-derived exosomes (BMSC-Exo) enriched with miR-125b (BMSC-Exo-125b) significantly enhanced cardiomyocyte viability and reduced apoptosis (indicated by a lower apoptotic ratio, Bax and caspase-3 expression, and elevated Bcl-2 levels) by downregulating sirtuin-7 (SIRT7), thereby inhibiting the intrinsic apoptotic pathway [12]. Similarly, human umbilical cord MSC-derived exosomes (hucMSC-Exo) restored miR-19a expression in ischemic myocardium and hypoxic cardiomyocytes [34]. Both native and miR-19a-enriched hucMSC-Exo reduced apoptosis (decreased expression of Bax and cleaved caspase-3, and increased Bcl-2 expression) by delivering miR-19a that inhibited SRY-box transcription factor-6 (SOX6), leading to Akt activation and suppression of the JNK3/caspase-3 apoptotic axis [34].

Consistent with these findings, mESC-derived exosomes transporting miR-294 enhanced cardiomyocyte survival and promoted CPC proliferation and differentiation, thereby contributing to myocardial regeneration and recovery [36]. In H/R-injured H9c2 cardiomyocytes, BMSC-Exo reduced apoptosis via upregulation of HAND2-AS1, which inhibited miR-17-5p and activated mitofusin-2 (Mfn2), suppressing mitochondrial-mediated cell death [3]. ADSC-Exo delivering miR-205 exerted similar anti-apoptotic effects by lowering the apoptotic rate and cleaved caspase-3 levels [37]. Furthermore, ischemic myocardial targeting peptide (IMTP)-hucMSC exosomes were shown to exert protective effects in I/R-injured myocardium, significantly lowering the expression of Bax, FasL, and caspase-3. Transcriptomic analyses identified gap junction alpha-1 (GJA1), high-mobility group box-1 (HMGB1), and PTEN as key molecular targets mediating these effects, primarily through modulation of the PI3K/Akt and apoptosis pathways [35].

### 3.6. Anti-Inflammatory Mechanisms of MSC-Derived Exosomes

MSC-derived exosomes exhibited potent anti-inflammatory effects, mitigating myocardial ischemic injury by suppressing pro-inflammatory cytokine production, modulating macrophage polarization, and inhibiting key inflammatory signaling pathways. These effects were primarily mediated by exosomal miRNAs such as miR-25-3p, miR-19a, and miR-125b, through regulation of the JAK2/STAT3 and PI3K/Akt signaling pathways [3,4,12,34,35].

BMSC-Exo-125b reduced myocardial inflammation by decreasing the expression of interleukin (IL)-1β, IL-6, and tumor necrosis factor-alpha (TNF-α), while limiting inflammatory cell infiltration in cardiac tissue. This anti-inflammatory response was associated with downregulation of SIRT7, which concurrently suppressed apoptotic signaling [12]. Similarly, BMSC-Exo carrying miR-25-3p (BMSC-Exo-25-3p) alleviated myocardial I/R injury by suppressing JAK2/STAT3 signaling, thereby inhibiting M1 macrophage polarization (indicated by reduced inducible nitric oxide synthase [iNOS], IL-1β, and IL-6 expression) and promoting M2 macrophage polarization (indicated by increased CD163, IL-10, and arginase-1 [Arg-1] expression). Consistent effects were observed in H/R-injured H9c2 cells and LPS-stimulated RAW 264.7 macrophages, confirming that exosome-mediated JAK2/STAT3 inactivation reduced pro-inflammatory cytokine release and supported anti-inflammatory macrophage phenotype [4].

HucMSC-Exo, particularly those enriched with miR-19a, also exhibited systemic anti-inflammatory activity in acute MI rats by reducing serum IL-1β and IL-18 levels [34]. In H/R-injured H9c2 cardiomyocytes, BMSC-Exo downregulated IL-1β, IL-6, and TNF-α through activation of the HAND2-AS1/miR-17-5p/Mfn2 regulatory axis, linking improved mitochondrial dynamics with reduced inflammatory signaling [3]. Furthermore, IMTP-hucMSC exosome treatment reduced pro-inflammatory cytokine (TNF-α, monocyte chemoattractant protein-1 [MCP-1], IL-1β, and IL-6) release and promoted M2 macrophage polarization [35].

### 3.7. Antioxidant and Mitochondrial-Protective Mechanisms of MSC-Derived Exosomes

MSC-derived exosomes demonstrated significant antioxidant and mitochondrial-protective effects by enhancing antioxidant enzyme activity, suppressing reactive oxygen species (ROS) generation, and preserving mitochondrial integrity, a process closely linked to attenuation of cardiomyocyte senescence following ischemic stress [3,35,37]. In H/R-injured cardiomyocytes, BMSC-Exo decreased oxidative stress, evidenced by decreased MDA levels and increased SOD activity. These effects were accompanied by the upregulation of the HAND2-AS1/miR-17-5p/Mfn2 axis, which collectively mitigated mitochondrial dysfunction, oxidative stress, inflammation, and apoptosis, thereby enhancing cardiomyocyte survival [3]. Similarly, ADSC-Exo attenuated oxidative injury in H/R-exposed NRCMs by reducing intracellular ROS generation [37]. In addition, treatment with IMTP-hucMSC exosomes decreased myocardial MDA levels in I/R rats, and in H/R-injured cardiomyocytes, lowered ROS and MDA while increasing SOD activity [35].

### 3.8. Angiogenesis and Myocardial Repair Mechanisms of MSC-Derived Exosomes

MSC-derived exosomes markedly enhanced angiogenesis and myocardial repair following ischemic injury by promoting endothelial cell activation, neovascularization, and CPC proliferation and differentiation. These effects were mediated primarily through the delivery of pro-angiogenic miRNAs, such as miR-205 and the miR-290 family, and through the upregulation of angiogenic signaling molecules including VEGF, HIF-1α, and CD31 [35,36,37]. In addition to promoting angiogenesis, several studies reported reduced myocardial fibrosis and improved extracellular matrix organization, indicating that MSC-derived exosomes also exert indirect anti-fibrotic effects during post-ischemic remodeling [35,36,37].

In a murine model of acute MI, treatment with mESC-Exo increased myocardial capillary density and enhanced cardiomyocyte cycling at four weeks post-AMI. Within the myocardium, CPCs exhibited increased survival, proliferation, and metabolic activity, with elevated expression of cardiomyocyte and endothelial cell markers and improved tube formation capacity. Furthermore, transplantation of mESC-Exo-pretreated CPCs led to enhanced neovascularization and cardiomyocyte formation eight weeks post-AMI, indicating that exosomes promote long-term myocardial regeneration. These effects were largely attributed to miR-294, a member of the miR-290 family, which stimulated CPC proliferation and survival [36].

Similarly, in acute MI mice, ADSC-Exo promoted angiogenesis in the infarcted myocardium, as evidenced by increased protein expression of CD31, HIF-1α, and VEGF. In H/R-injured HMEC-1, ADSC-Exo treatment upregulated HIF-1α, VEGF, and miR-205 expression, while inhibition of miR-205 attenuated these angiogenic effects, confirming its role as a key mediator of ADSC-Exo-induced vascular regeneration [37]. Consistent findings were observed in IMTP-hucMSC exosome-treated models, where therapy enhanced arteriolar and capillary regeneration within the infarct border zone of I/R rats and increased tube formation in HUVECs, further supporting their pro-angiogenic potential [35].

## 4. Discussion

This systematic review consolidates the growing body of preclinical evidence demonstrating that MSC-derived exosomes exert robust cardioprotective effects against myocardial ischemic injury through a complex interplay of anti-apoptotic, anti-inflammatory, antioxidant, angiogenic, and myocardial repair mechanisms. The included studies consistently report significant improvements in cardiac function, reduction of infarct size, and preservation of myocardial structure following exosome treatment in both in vivo acute MI and I/R models, as well as in vitro H/R systems. These effects were reproducibly observed across rodent and cell-based models, indicating that exosome-mediated cardioprotection is both robust and mechanistically multifactorial. The predominant molecular pathways involved include the PI3K/Akt, JAK2/STAT3, and JNK3/caspase-3 cascades, complemented by contributions from antioxidant and angiogenic signaling networks.

Apoptosis is a central pathological process in myocardial ischemic injury, primarily triggered by mitochondrial dysfunction, calcium overload, and oxidative stress [30]. Several studies have demonstrated that MSC-derived exosomes attenuate cardiomyocyte apoptosis by targeting key regulators of the intrinsic apoptotic pathway. The PI3K/Akt pathway has emerged as a critical survival axis modulated by multiple exosomal miRNAs. Activation of PI3K catalyzes the phosphorylation of Akt, which in turn suppresses pro-apoptotic proteins such as Bax, caspase-3, and FasL [41]. In addition, Akt activation inhibits glycogen synthase kinase-3β (GSK3β), thereby preventing the opening of the mitochondrial permeability transition pore that leads to cytochrome c release and apoptosome formation [42]. In parallel, Akt enhances the nuclear translocation of NF-κB and cyclic-AMP response element binding (CREB), promoting transcription of anti-apoptotic genes such as Bcl-2 [42]. Together, these molecular events reprogram cardiomyocytes toward a pro-survival phenotype, enabling them to withstand oxidative and metabolic stress during reperfusion.

For instance, hucMSC-Exo delivering miR-19a activated Akt by directly inhibiting SOX6, which otherwise represses PI3K/Akt signaling [34]. This cascade ultimately suppressed the JNK3/caspase-3 axis, a downstream executor of apoptosis, thereby maintaining mitochondrial integrity and enhancing cardiomyocyte viability. Similarly, BMSC-Exo enriched with miR-125b inhibited apoptosis via downregulation of SIRT7 [12]. SIRT7 is a nicotine adenine dinucleotide (NAD^+^)-dependent deacetylase that promotes stress-induced apoptotic gene expression [43]. Its suppression decreased Bax and caspase-3 activation while restoring Bcl-2 levels, reflecting the canonical anti-apoptotic effect of Akt activation [44]. Moreover, ADSC-Exo carrying miR-205 further demonstrated Akt-mediated cytoprotection by reducing cleaved caspase-3 levels and apoptotic rates in H/R-injured cardiomyocytes [37]. Collectively, these studies reinforce that MSC-exosomal miRNAs act upstream of PI3K/Akt signaling, redirecting cellular fate from apoptosis toward survival through coordinated gene silencing of apoptosis-promoting factors.

The HAND2-AS1/miR-17-5p/Mfn2 regulatory network exemplifies how MSC-derived exosomes couple mitochondrial homeostasis to anti-apoptotic protection. HAND2-AS1, a lncRNA highly expressed in exosomes derived from bone marrow MSCs, inhibits miR-17-5p, which normally suppresses Mfn2 [3]. Mfn2 is a key protein governing mitochondrial fusion and bioenergetic efficiency [45]. By relieving miR-17-5p-mediated repression, HAND2-AS1 promotes Mfn2 upregulation, leading to improved mitochondrial connectivity and reduced cytochrome c release from damaged mitochondria [46]. This exosome-mediated modulation of mitochondrial dynamics is particularly significant, as Mfn2 not only maintains mitochondrial morphology but also stabilizes the endoplasmic reticulum-mitochondria contact sites, which regulate calcium flux and apoptotic signaling [46]. Through this mechanism, exosomes restore mitochondrial membrane potential, prevent cardiolipin oxidation, and preserve ATP generation capacity, thus directly supporting energy homeostasis during reperfusion stress [47]. This mechanism not only suppresses apoptotic cascades but also alleviates oxidative stress and inflammation, emphasizing the multifunctional role of mitochondrial stabilization in exosome-mediated cardioprotection.

MSC-derived exosomes also attenuate inflammation by suppressing pro-inflammatory signaling in cardiomyocytes and macrophages and by promoting M2 macrophage polarization, which facilitates tissue repair. The JAK2/STAT3 pathway has emerged as a pivotal inflammatory cascade targeted by exosomal miRNAs [4]. STAT3 is a transcriptional regulator that drives M1 polarization through IL-6 and TNF-α production [48]. Its inhibition shifts macrophages toward an M2 phenotype characterized by high Arg-1, IL-10, and CD163 expression. This phenotypic switch not only suppresses pro-inflammatory cytokine release but also promotes efferocytosis and matrix remodeling that are essential for post-injury recovery [49]. BMSC-Exo carrying miR-25-3p inhibited JAK2/STAT3 activation, suppressing M1 macrophage polarization, while simultaneously inducing M2 polarization [4]. This shift from a pro-inflammatory to an anti-inflammatory macrophage phenotype reduces local cytokine release and limits secondary myocardial damage. Such findings align with previous studies showing that JAK2/STAT3 suppression attenuates cytokine storms and leukocyte infiltration during reperfusion injury [50].

Notably, hucMSC-Exo enriched with miR-19a also suppressed serum and cardiac IL-1β and IL-18 levels in MI rats, demonstrating that exosomal cargo can modulate both systemic and local inflammatory responses [34]. The convergence of JAK2/STAT3 inhibition and macrophage reprogramming establishes MSC-derived exosomes as key regulators of immune balance in the post-ischemic myocardium. Concurrently, BMSC-Exo-125b decreased cardiac IL-1β, IL-6, and TNF-α levels via SIRT7 downregulation [12], highlighting the overlap between anti-apoptotic and anti-inflammatory signaling. Interestingly, SIRT7 downregulation indirectly inhibits NF-κB activation by reducing p65 acetylation, thereby limiting transcription of pro-inflammatory genes [51]. This crosstalk between sirtuin regulation and cytokine suppression positions SIRT7 as a central control node integrating inflammation and apoptosis.

Oxidative stress, resulting from excessive ROS production during reperfusion, exacerbates myocardial injury by oxidizing lipids and proteins and activating apoptotic pathways [30]. MSC-derived exosomes mitigate this oxidative burst through both direct enhancement of antioxidant enzyme activity and indirect modulation of mitochondrial function. Treatment with BMSC-Exo markedly reduced MDA levels while enhancing SOD activity, reflecting reduced lipid peroxidation and improved redox balance [3]. The HAND2-AS1/miR-17-5p/Mfn2 pathway again plays a central role, as Mfn2 upregulation with BMSC-Exo treatment restores mitochondrial electron transport efficiency and minimizes ROS leakage [3]. Similarly, ADSC-derived exosomes reduced intracellular ROS accumulation in cardiomyocytes exposed to H/R injury [37], suggesting conserved antioxidant effects across MSC sources. In I/R rats, IMTP-hucMSC exosomes lowered myocardial MDA and increased SOD [35], further demonstrating that MSC-derived exosomes augment endogenous antioxidant defense mechanisms. Mechanistically, Akt activation enhances transcription of antioxidant genes such as nuclear factor erythroid 2-related factor 2 (Nrf2), HO-1, and SOD, linking anti-apoptotic and antioxidant signaling [52]. This coordinated regulation of redox homeostasis and mitochondrial preservation positions MSC-derived exosomes as a dynamic defense system against reperfusion-induced oxidative damage.

Beyond cytoprotection, MSC-derived exosomes actively contribute to angiogenesis and tissue regeneration. Angiogenesis is crucial for restoring oxygen supply and promoting structural recovery following ischemic injury [53]. Several studies revealed that MSC-derived exosomes enhance endothelial proliferation, migration, and tube formation. Exosomal delivery of pro-angiogenic miRNAs such as miR-205 and members of the miR-290 family (miR-291, miR-294, miR-295) promoted neovascularization and endothelial activation via upregulation of VEGF, HIF-1α, and CD31 [35,36,37]. In mESC-Exo-treated MI mice, increased myocardial capillary density and enhanced CPC proliferation were observed four weeks post-MI. The delivery of miR-294 stimulated CPC survival and differentiation toward cardiomyocyte and endothelial lineages, while transplantation of mESC-Exo-pretreated CPCs led to persistent neovascularization and de novo cardiomyocyte formation eight weeks post-MI [36]. These findings suggest that exosomal cargo not only prevents cardiomyocyte death but also reactivates endogenous regenerative programs within the myocardium.

Similarly, ADSC-Exo enhanced vascular regeneration in infarcted myocardium by increasing HIF-1α, VEGF, and CD31 expression, effects that were abrogated by miR-205 inhibition, confirming its functional necessity [37]. The IMTP-hucMSC exosomes further extended these effects by promoting arteriolar and capillary regeneration in the infarct border zone and stimulating tube formation in HUVECs [35]. Together, these data indicate that MSC-derived exosomes can restore perfusion and microvascular density, thereby enhancing oxygen delivery to the recovering myocardium.

**Figure 3:**
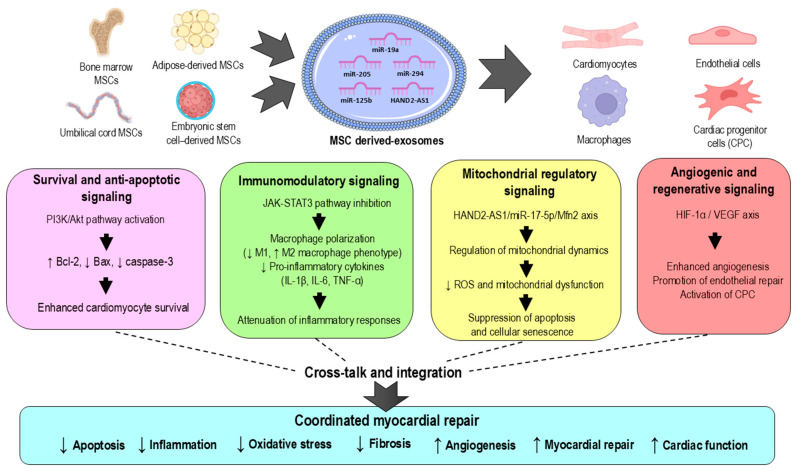
Mechanistic overview of mesenchymal stem cell (MSC)-derived exosome-mediated cardioprotection in myocardial ischemic injury. MSC-derived exosomes originating from bone marrow, adipose tissue, umbilical cord, or embryonic stem cell-derived MSCs are internalized by cardiomyocytes, endothelial cells, macrophages, and cardiac progenitor cells. Exosomal microRNAs and long non-coding RNAs modulate interconnected signaling pathways, including PI3K/Akt, JAK2/STAT3, HAND2-AS1/miR-17-5p/Mfn2, and HIF-1α/VEGF, thereby regulating cell survival, inflammation, mitochondrial dynamics, and vascular regeneration. Dotted lines indicate potential cross-talk and indirect regulatory interactions among signaling pathways. Collectively, these mechanisms attenuate apoptosis, inflammation, oxidative stress, and fibrosis while promoting angiogenesis, myocardial repair, and functional recovery, highlighting the therapeutic potential of MSC-derived exosomes as a cell-free strategy for myocardial ischemic injury.

Collectively, the findings across these studies reveal that MSC-derived exosomes exert cardioprotection through interconnected and mutually reinforcing mechanisms rather than isolated molecular events (Figure 3). Key pathways, particularly PI3K/Akt and JAK2/STAT3, function as central regulatory nodes that integrate exosomal miRNA and lncRNA signals to coordinate apoptosis suppression, inflammatory modulation, oxidative stress control, and angiogenic responses. In parallel, mitochondrial stabilization through the HAND2-AS1/miR-17-5p/Mfn2 axis and angiogenic activation via the miR-205/HIF-1α/VEGF pathway further enhance tissue repair. This coordinated signaling response likely explains the consistent cardioprotective effects observed across diverse experimental models and highlights the multifaceted therapeutic potential of MSC-derived exosomes.

Although no study demonstrated a high risk of bias, the consistent lack of reporting regarding blinding of caregivers and outcome assessors represents a potential source of performance and detection bias. In preclinical experimental studies, absence of blinding may lead to inadvertent overestimation of treatment effects. Additionally, small sample sizes and the absence of detailed power calculations in several studies may further limit the precision and robustness of the reported outcomes. Therefore, while the mechanistic findings appear consistent across models, the overall strength of evidence should be interpreted with appropriate caution. Future studies should adhere to rigorous experimental design standards, including transparent reporting of randomization procedures, blinding practices, and sample size justification, to enhance reproducibility and translational reliability.

## 5. Limitations and Future Directions

Although preclinical evidence strongly supports the cardioprotective potential of MSC-derived exosomes, several challenges must be addressed before their successful clinical translation. Substantial heterogeneity in exosome source, dosing regimens, administration routes, and timing complicates cross-study comparability and hinders the establishment of standardized therapeutic protocols. The absence of validated potency assays for exosome preparations further limits reproducibility and scalability for clinical-grade manufacturing. Importantly, none of the included studies reported major adverse effects associated with MSC-derived exosome administration; however, systematic safety assessments, including immunogenicity, off-target biodistribution, and long-term toxicity, remain limited. Moreover, most existing data are derived from rodent models, which, although invaluable for mechanistic insight, do not fully capture the structural, electrophysiological, and immunological complexity of the human myocardium.

Exosome source may influence therapeutic efficacy, as bone marrow-, adipose-, umbilical cord-, and embryonic stem cell-derived MSCs exhibit distinct cargo profiles and regenerative capacities. However, direct comparative studies are lacking, and it remains unclear whether specific MSC sources confer superior cardioprotective potency. Considerable variability also exists in dosing strategies and timing of administration, with delivery performed at pre-ischemic, reperfusion, and post-infarction stages. Systematic dose–response and pharmacokinetic studies are therefore necessary to define effective and safe therapeutic windows.

Route of administration further affects bioavailability. Intramyocardial injection enables localized delivery but is invasive, whereas intravenous administration is less invasive yet may result in pulmonary or hepatic sequestration, reducing myocardial bioavailability. Strategies to enhance cardiac homing, including surface modification and bioengineering approaches, merit further investigation. Long-term safety, biodistribution, and regulatory scalability will require rigorous evaluation in large-animal models and establishment of Good Manufacturing Practice-compliant production and quality control standards.

Moving forward, priority should be given to standardized isolation protocols, validated potency assays, and consistent reporting of dosing metrics. Large-animal studies incorporating pharmacokinetic and biodistribution analyses will be essential to bridge the translational gap. Integrative multi-omics profiling may further define the functional cargo responsible for cardioprotection and identify synergistic molecular interactions. In parallel, advances in exosome bioengineering and targeting strategies may enhance myocardial retention and therapeutic precision. Addressing these priorities through coordinated multidisciplinary efforts will be critical to advancing MSC-derived exosomes toward safe and effective clinical application.

## 6. Conclusions

This systematic review provides consolidated mechanistic evidence that MSC-derived exosomes exert robust cardioprotective effects in myocardial ischemic injury through coordinated modulation of apoptosis, inflammation, oxidative stress, angiogenesis, and myocardial repair. By simultaneously modulating apoptosis, inflammation, oxidative stress, angiogenesis, and myocardial repair through interconnected pathways, most notably PI3K/Akt, JAK2/STAT3, HAND2-AS1/miR-17-5p/Mfn2, and HIF-1α/VEGF, these nanoscale vesicles restore cardiomyocyte viability and function, preserve mitochondrial integrity, and promote neovascularization following ischemic insult. Their multifactorial bioactivity, low immunogenicity, and potential for targeted delivery position MSC-derived exosomes as an attractive therapy for ischemic heart disease. Future translational efforts integrating standardized manufacturing, mechanistic profiling, and advanced delivery systems will be crucial to harness their full potential as a clinically viable, next-generation nanomedicine for cardiac repair.

## Figures and Tables

**Table 1:** Summary of the cardioprotective mechanisms of MSC-derived exosomes in myocardial ischemic injury.

Exosome Source	Experimental Model and Exosome Treatment	Timing of Exosome Administration	Key Findings	Proposed Mechanism	Author (Year)
Bone marrow MSCs (BMSCs) isolated from the femur and tibia of Sprague–Dawley (SD) rats	In vivo: SD rat I/R model (left anterior descending (LAD) coronary artery ligation, 30 min ischemia + 2 h reperfusion); intramyocardial injection of 50 µg of BSMC-Exo carrying miR-125b (BMSC-Exo-125b) In vitro: cardiomyocytes isolated from I/R rats; 50 µg BMSC-Exo-125b for 48 h	In vivo: At reperfusion onset In vitro: Post-injury ex vivo treatment	↓ Infarct size ↓ Inflammation (↓ IL-1β, IL-6 and TNF-α) ↓ Inflammatory cell infiltration ↑ Cardiomyocyte viability ↓ Cardiomyocyte apoptosis (↓ apoptotic ratio, Bax, and caspase-3; ↑ Bcl-2) Improved cardiac function (↑ LVEF, LVFS, LVSP and ± dp/d*t*_max_; ↓ LVESD, LVEDD and LVEDP) ↓ SIRT7 mRNA and protein expression in the myocardium	BMSC-Exo-125b protects against MIRI by downregulating SIRT7, thereby inhibiting inflammation and apoptosis.	Chen et al. (2020) [12]
BMSCs isolated from SD rats	In vivo: SD rat I/R model (LAD coronary artery ligation for 30 min + 2 h reperfusion); tail vein injection of 100 µg/kg BSMC-Exo carrying miR-25-3p (BMSC-Exo-25-3p) In vitro: hypoxia/reoxygenation (H/R)-induced H9c2 cardiomyocytes; lipopolysaccharide (LPS)-stimulated RAW 264.7 macrophages; co-culture model (100 µg BMSC-Exo-25-3p for 48 h)	In vivo: Pre-ischemic (2 h before LAD ligation) In vitro: At reoxygenation onset	↓ Myocardial infarct size ↓ Malignant arrhythmias ↓ Serum CK and LDH ↓ CK and LDH enzymatic activity ↓ M1 macrophage markers (iNOS, IL-1β, IL-6) ↑ M2 macrophage markers (CD163, IL-10, Arg-1) ↓ JAK2/STAT3 signaling	BMSC-Exo-25-3p attenuates MIRI by inhibiting JAK2/STAT3 signaling, thereby suppressing M1 macrophage polarization and pro-inflammatory cytokine release.	Du et al. (2024) [4]
Human umbilical cord MSCs (hucMSCs)	In vivo: SD rat acute myocardial infarction (AMI) model (LAD coronary artery ligation); tail-vein injection of 400 µg/g hucMSC-Exo In vitro: hypoxic H9c2 cardiomyocytes; 24 h treatment (dose not specified)	In vivo: During ischemia (immediately after LAD ligation) In vitro: During hypoxia	↓ Inflammation (↓ IL-1β, IL-18) ↓ Myocardial injury (↓ cTnT, CK-MB and LDH) ↓ Infarct size Improved cardiac morphology ↓ Apoptosis (↓ apoptotic ratio, Bax, and cleaved caspase-3; ↑ Bcl-2) Improved cardiac function (↑ LVEF and LVFS; ↓ LVESD and LVEDD) ↑ Cardiomyocyte proliferation and migration	hucMSC-exo protects cardio myocytes from AMI-induced injury by delivering miR 19a which inhibits SOX6, activates Akt signaling, and suppresses the JNK3/caspase-3 apoptotic axis.	Huang et al. (2020) [34]
Murine embryonic stem cells (mESCs) isolated from C57Bl/6 mice	In vivo: Mouse AMI model (LAD coronary artery ligation); intramyocardial injection of mESC-Exo or mESC-Exo-pretreated CPCs (dose not specified) In vitro: Hydrogen peroxide (H_2_O_2_)-induced cardiac progenitor cells (CPCs) and H9c2 cardiomyocytes; 16 h exposure to mESC-Exo (dose not specified)	In vivo: During ischemia (immediately after LAD ligation) In vitro: Co-treatment during H_2_O_2_-induced oxidative stress	Improved cardiac function (↑ LVEF, LVFS and LVWM; ↓ LVESD) ↓ Myocardial infarct size, apoptosis and fibrosis ↑ Myocardial capillary density and neovascularization ↑ Cardiomyocyte cycling ↑ CPC survival, proliferation, tube formation ability and cardiac differentiation mESC-Exo are highly enriched with miR-290 family including miR-291, miR-294 and miR-295. miR-294 promoted proliferation and survival of CPCs.	mESC-Exo deliver miR-294 to the heart, promoting CPC survival, proliferation, and cardiomyogenic differentiation that enhances myocardial regeneration.	Khan et al. (2015) [36]
BMSCs (source not specified)	H/R-induced H9c2 cardiomyocytes; 5 µg BMSC-Exo for 48 h	At reoxygenation onset	↑ Cell viability ↓ Apoptotic cells ↓ Inflammation (↓ IL-1β, IL-6, TNF-α) ↓ Oxidative stress (↓ MDA and LDH levels, ↑ SOD activity) ↑ Expression of lncRNA HAND2-AS1	BMSC-Exo activates the HAND2-AS1/miR-17-5p/Mfn2 axis, reducing apoptosis, oxidative stress, and inflammation.	Li et al. (2023) [3]
Adipose tissue-derived MSCs (ADSCs) isolated from C57Bl/6 mice inguinal subcutaneous fat	In vivo: C57Bl/6 mouse AMI model (LAD coronary artery ligation); intramyocardial injection of 100 µg ADSC-Exo In vitro: H/R-injured neonatal rat cardiomyocytes (NRCMs) and human microvascular endothelial cells (HMEC-1); 24 h exposure to ADSC-Exo (dose not specified)	In vivo: During ischemia (25 min after LAD ligation) In vitro: At reoxygenation onset	Improved cardiac function (↑ LVEF and LVFS) ↓ Myocardial fibrosis and infarct area ↑ Neovessel formation (↑ CD31, HIF-1α and VEGF) ↓ Cardiomyocyte apoptosis ↓ Cardiomyocyte ROS production ↑ Endothelial cell viability and proliferation ↓ Endothelial Apoptosis (↓ cleaved caspase-3) ↑ Angiogenic signaling (↑ HIF-1α and VEGF) ↑ miR-205 expression	ADSC-Exo deliver miR-205, promoting angiogenesis and inhibiting apoptosis.	Wang et al. (2023) [37]
hucMSC-derived exosomes	In vivo: SD rat I/R model (LAD ligation for 30 min followed by reperfusion); tail-vein injection of 400 µg ischemic myocardial targeting peptide (IMTP)-hucMSC-Exo In vitro: H/R-induced human embryonic stem cell (hESC)-derived cardiomyocytes and HUVECs; 100 µg IMTP-hucMSC-Exo for 6 h	In vivo: At reperfusion onset In vitro: At reoxygenation onset	↓ inflammation (↓ TNF-α, MCP-1, IL-1β, IL-6 and M1 macrophage, ↑ M2 macrophage) ↓ Oxidative stress (↓ ROS and MDA, ↑ SOD) ↓ Apoptosis (↓ apoptotic rate, Bax, caspase-3 and FasL) ↓ Infarct size Improved cardiac histopathology (↓ myocardial necrosis, fibrosis, disordered arrangement, and inflammatory cell infiltration) ↑ Angiogenesis and myocardial repair Improved cardiac function (↑ LVEF and LVFS, ↓ LVESD and LVEDD)	IMTP-hucMSC-Exo promotes myocardial repair by attenuating oxidative stress, inflammation, apoptosis and fibrosis. The key genes involved include GJA1, HMGB1 and PTEN, which primarily modulate the PI3K-Akt signaling and apoptosis pathways.	Wang et al. (2025) [35]

Abbreviations: ↑ = increase; ↓ = decrease; ADSC = adipose-derived mesenchymal stem cell; AKT = protein kinase B; AMI = acute myocardial infarction; Arg-1 = arginase-1; Bax = Bcl-2-associated X protein; Bcl-2 = B-cell lymphoma-2; BMSC = bone marrow mesenchymal stem cell; BMSC-Exo = BMSC-derived exosomes; BrdU = bromodeoxyuridine; c-kit = tyrosine-protein kinase Kit (CD117); CD31 = platelet endothelial cell adhesion molecule-1; CD163 = cluster of differentiation 163; CK = creatine kinase; CK-MB = creatine kinase-MB; CPC = cardiac progenitor cell; cTnT = cardiac troponin T; dp/dtmax (+/−) = maximum rate of LV pressure rise/fall; Exo = exosome(s); FasL = Fas ligand; GFP = green fluorescent protein; GJA1 = gap junction alpha-1 (connexin-43); H_2_O_2_ = hydrogen peroxide; HAND2-AS1 = heart- and neural crest derivatives expressed 2 antisense RNA 1; hESC = human embryonic stem cell; HIF-1α = hypoxia-inducible factor-1 alpha; HMEC-1 = human microvascular endothelial cell line-1; HMGB1 = high-mobility group box-1; H/R = hypoxia/reoxygenation; HUVEC = human umbilical vein endothelial cell; hucMSC = human umbilical cord mesenchymal stem cell; I/R = ischemia/reperfusion; IL-1β = interleukin-1 beta; IL-10 = interleukin-10; IL-6 = interleukin-6; IMTP = ischemic myocardial targeting peptide; iNOS = inducible nitric oxide synthase; JAK2 = Janus kinase-2; JNK3 = c-Jun N-terminal kinase-3; LAD = left anterior descending (coronary artery); LDH = lactate dehydrogenase; lncRNA = long non-coding RNA; LVEDD = left ventricular end-diastolic diameter; LVEDP = left ventricular end-diastolic pressure; LVEF = left ventricular ejection fraction; LVESD = left ventricular end-systolic diameter; LVFS = left ventricular fractional shortening; LVSP = left ventricular systolic pressure; LVWM = left ventricular wall motion; MCP-1 = monocyte chemoattractant protein-1; MDA = malondialdehyde; Mfn2 = mitofusin-2; miR = microRNA; NRCM = neonatal rat cardiomyocyte; PI3K = phosphoinositide 3-kinase; PTEN = phosphatase and tensin homolog; RAW 264.7 = mouse macrophage cell line RAW 264.7; ROS = reactive oxygen species; SD = Sprague–Dawley; SIRT7 = sirtuin-7; SOD = superoxide dismutase; SOX6 = SRY-box transcription factor-6; STAT3 = signal transducer and activator of transcription-3; TNF-α = tumor necrosis factor-alpha; VEGF = vascular endothelial growth factor.

## Data Availability

No new data were created or analyzed in this study. Additionally, the research protocol is publicly accessible on the International Platform of Registered Systematic Review and Meta-analysis Protocols (INPLASY) database under registration number INPLASY2024110023 and can be found at https://inplasy.com/inplasy-2024-11-0023/ (accessed on 15 January 2026). Further inquiries can be directed to the corresponding author.

## Supplementary Materials

The following supporting information can be downloaded at: https://www.mdpi.com/article/10.3390/pharmaceutics18030346/s1, PRISMA 2020 Checklist.

## Abbreviations

The following abbreviations are used in this manuscript:

ADSC	Adipose-derived mesenchymal stem cell
Akt	Protein kinase B
AMI	Acute myocardial infarction
Arg-1	Arginase-1
Bax	Bcl-2-associated X protein
Bcl-2	B-cell lymphoma-2
BMSC	Bone marrow mesenchymal stem cell
BMSC-Exo	BMSC-derived exosomes
BrdU	Bromodeoxyuridine
CD31	Platelet endothelial cell adhesion molecule-1
CD163	Cluster of differentiation 163
c-kit	Tyrosine-protein kinase Kit (CD117)
CK	Creatine kinase
CK-MB	Creatine kinase-MB
CPC	Cardiac progenitor cell
cTnT	Cardiac troponin T
dp/dt_max_ (+/−)	Maximum rate of left ventricular pressure rise/fall
Exo	Exosome(s)
FasL	Fas ligand
GFP	Green fluorescent protein
GJA1	Gap junction alpha-1 (connexin-43)
H_2_O_2_	Hydrogen peroxide
HAND2-AS1	Heart- and neural crest derivatives expressed 2 antisense RNA 1
hESC	Human embryonic stem cell
HIF-1α	Hypoxia-inducible factor-1 alpha
HMEC-1	Human microvascular endothelial cell line-1
HMGB1	High-mobility group box-1
H/R	Hypoxia/reoxygenation
hucMSC	Human umbilical cord mesenchymal stem cells
HUVEC	Human umbilical vein endothelial cells
I/R	Ischemia/reperfusion
IL-1β	Interleukin-1 beta
IL-6	Interleukin-6
IL-10	Interleukin-10
IMTP	Ischemic myocardial targeting peptide
iNOS	Inducible nitric oxide synthase
JAK2	Janus kinase-2
JNK3	c-Jun N-terminal kinase-3
LAD	Left anterior descending coronary artery
LDH	Lactate dehydrogenase
lncRNA	Long non-coding RNA
LVEDD	Left ventricular end-diastolic diameter
LVEDP	Left ventricular end-diastolic pressure
LVEF	Left ventricular ejection fraction
LVESD	Left ventricular end-systolic diameter
LVFS	Left ventricular fractional shortening
LVSP	Left ventricular systolic pressure
LVWM	Left ventricular wall motion
MCP-1	Monocyte chemoattractant protein-1
MDA	Malondialdehyde
Mfn2	Mitofusin-2
miR	MicroRNA
NRCM	Neonatal rat cardiomyocyte
PI3K	Phosphoinositide 3-kinase
PTEN	Phosphatase and tensin homolog
RAW 264.7	Mouse macrophage cell line RAW 264.7
ROS	Reactive oxygen species
SD	Sprague–Dawley
SIRT7	Sirtuin-7
SOD	Superoxide dismutase
SOX6	SRY-box transcription factor-6
STAT3	Signal transducer and activator of transcription-3
TNF-α	Tumor necrosis factor-alpha
VEGF	Vascular endothelial growth factor
